# Supplementary material for: Genomic and Metabolic Characterization of Plant Growth-Promoting Rhizobacteria Isolated from Nodules of Clovers Grown in Non-Farmed Soil
Source: Int J Mol Sci. 2023 Nov 23;24(23):16679. doi: 10.3390/ijms242316679 (PMC10706249; doi:10.3390/ijms242316679)
Supplement: Supplementary file 1 [file ijms-24-16679-s001.zip › ijms-2710802-supplementary.pdf]

Supplementary Data for

Genomic and metabolic characterization of plant growth-promoting rhizobacteria isolated from nodules of clover grown in non-farmed soil

Magdalena Wójcik, Piotr Koper, Kamil Żebracki, Małgorzata Marczak, Andrzej Mazur*

Department of Genetics and Microbiology, Institute of Biological Sciences, Maria Curie-Skłodowska University, Akademicka 19 St., 20-033 Lublin, Poland

*Correspondence: andrzej.mazur@mail.umcs.pl (A.M.)

This PDF file includes:

Supplementary Tables S1 to S2
Supplementary Figures S1 to S5

Supplementary Table S1. Sequencing data processing results.

Clover rhizosphere microbiota	Sample name	Input sequences	Sequences after preprocessing	Sequences after chimera removal	Sequences assigned to OTUs	Sequences assigned to taxa
Non-farmed soil	A1	142,741	142,728	142,202	67,660	67,660
	A2	117,392	117,378	117,294	62,174	62,174
	A3	133,225	133,216	132,867	55,417	55,417
Agricultural soil	B1	154,950	154,933	154,578	68,491	68,491
	B2	126,259	126,253	125,933	53,292	53,292
	B3	135,907	135,896	135,600	67,351	67,351
	TOTAL	810,474	810,404	808,474	374,385	374,385

Supplementary Table S2. Comparison of alpha diversity indices within microbiota of soil rhizosphere of clover grown in non-farmed (A) and agricultural soil (B).

Clover rhizosphere microbiota	Sample	Chao1	Simpson	Shannon
Non-farmed soil	A1	42.2	0.892	3.242
	A2	51.8	0.898	3.302
	A3	41.2	0.890	3.222
Agricultural soil	B1	48.6	0.896	3.282
	B2	47.6	0.894	3.262
	B3	42.2	0.892	3.242

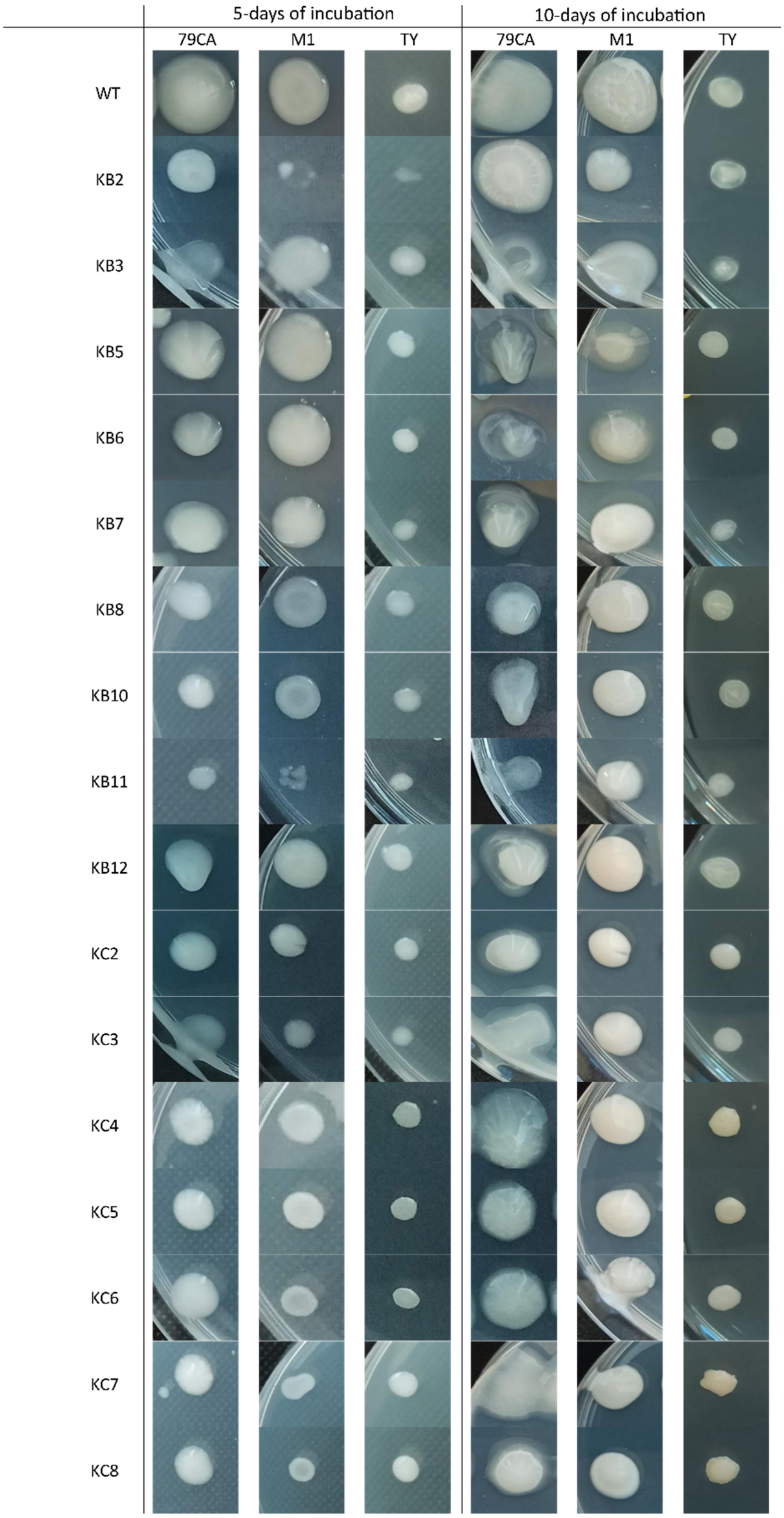

Supplementary Figure S1. Macroscopic characteristics of the nodule isolates derived from white (KB2-12) and red clover (KC2-8) growing in the buffer zone of Białowieża National Park compare with the wild type strain (*Rhizobium leguminosarum* bv. *trifolii* TA1 (marked as WT)) The strains were cultivated in solid 79CA, M1, and TY media. Bacterial suspensions in the bottom panel were washed and standardized to the same starting optical density. Photographs were taken 5 and 10 days post-inoculation (left and right, respectively).

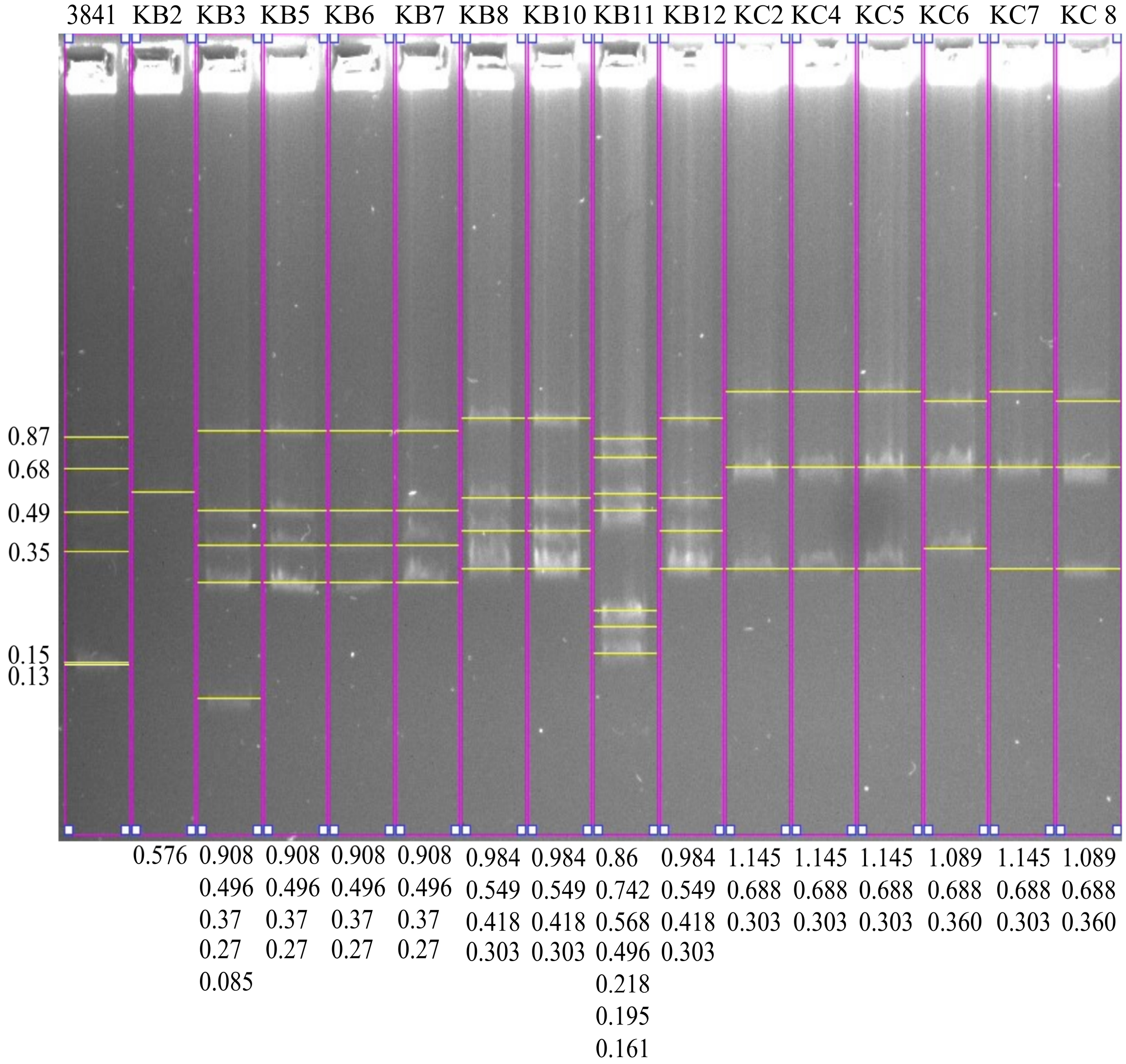

Supplementary Figure S2. Plasmid profiling of *Rhizobium* strains by the Eckhardt method. Lanes: 1: *Rhizobium leguminosarum* bv. *viciae* 3841^T (^{T}-type strain, ECR size marker); 2-17: *Rhizobium* isolates derived from white (KB2-12) and red clover (KC2-8) growing in the buffer zone of Białowieża National Park. The numbers on the left indicate the size of the replicons (in Mb) of the reference strain, which was the standard for determining the ECR size of the KB and KC strains The calculated size of KB and KC ECR (in Mb) determined on the basis of electrophoretic mobility relative to the reference strain, arranged from the largest to the smallest, were shown below the lanes.

***Rhizobium leguminosarum* KC5**	
Length: 7373243bp, Contigs: 81	
General Info	
Genome ID	384.1015
Genome Name	*Rhizobium leguminosarum* KC5
Taxonomy Info	
Taxon ID	384
Kingdom	Bacteria
Phylum	Proteobacteria
Class	Alphaproteobacteria
Order	Hyphomicrobiales
Family	*Rhizobiaceae*
Genus	*Rhizobium*
Species	*Rhizobium leguminosarum*
Genome Statistics	
Contigs	81
Genome Length	7373243
GC Content	60.731064
Contig L50	6
Contig N50	395773
Annotation Statistics	
tRNA	45
IRNA	3
CDS	7515
CDS Ratio	1.0192258
Hypothetical CDS	2144
Hypothetical CDS Ratio	0.38310048
PLFAM CDS	7078
PLFAM CDS Ratio	0.94184965
Genome Quality	
Coarse Consistency	99.5
Fine Consistency	95.8
CheckM Completeness	100
Genome Quality	Good

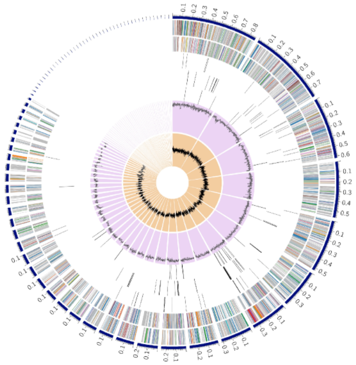

***Rhizobium leguminosarum* KC4**	
Length: 7341322bp, Contigs: 86	
General Info	
Genome ID	384.1014
Genome Name	*Rhizobium leguminosarum* KC4
Taxonomy Info	
Taxon ID	384
Kingdom	Bacteria
Phylum	Proteobacteria
Class	Alphaproteobacteria
Order	Hyphomicrobiales
Family	*Rhizobiaceae*
Genus	*Rhizobium*
Species	*Rhizobium leguminosarum*
Genome Statistics	
Contigs	86
Genome Length	7341322
GC Content	60.747234
Contig L50	7
Contig N50	310761
Annotation Statistics	
tRNA	45
rRNA	3
CDS	7478
CDS Ratio	1.0186176
Hypothetical CDS	2099
Hypothetical CDS Ratio	0.37951323
PLFAM CDS	7086
PLFAM CDS Ratio	0.94757956
Genome Quality	
Coarse Consistency	99.7
Fine Consistency	96.3
CheckM Completeness	100
Genome Quality	Good

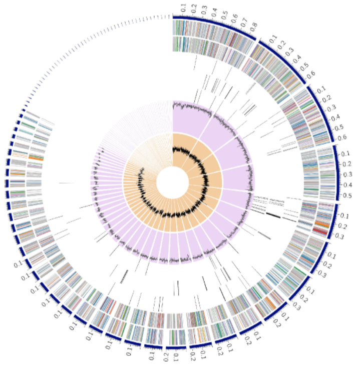

***Rhizobium leguminosarum* KB7**	
Length: 7098746bp, Contigs: 101	
General Info	
Genome ID	379.691
Genome Name	*Rhizobium leguminosarum* KB7
Taxonomy Info	
Taxon ID	379
Superkingdom	Bacteria
Phylum	Proteobacteria
Class	Alphaproteobacteria
Order	Hyphomicrobiales
Family	*Rhizobiaceae*
Genus	*Rhizobium*
Species	*Rhizobium leguminosarum*
Genome Statistics	
Contigs	101
Genome Length	7098746
GC Content	60.78547
Contig L50	11
Contig N50	221519
Annotation Statistics	
tRNA	46
rRNA	3
CDS	7202
CDS Ratio	1.0145454
Hypothetical CDS	1955
Hypothetical CDS Ratio	0.37017494
PLFAM CDS	6733
PLFAM CDS Ratio	0.9348792
Genome Quality	
Coarse Consistency	99.3
Fine Consistency	96.3
CheckM Completeness	100
Genome Quality	Good

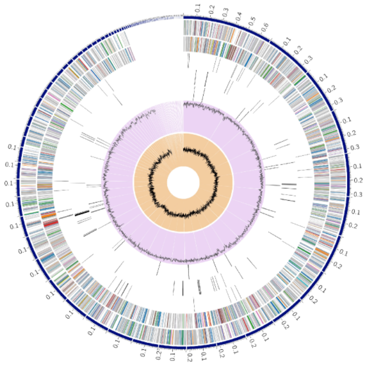

***Rhizobium leguminosarum* KB8**	
Length: 7412147bp, Contigs: 103	
General Info	
Genome ID	379.692
Genome Name	*Rhizobium leguminosarum* KB8
Taxonomy Info	
Taxon ID	379
Superkingdom	Bacteria
Phylum	Proteobacteria
Class	Alphaproteobacteria
Order	Hyphomicrobiales
Family	*Rhizobiaceae*
Genus	*Rhizobium*
Species	*Rhizobium leguminosarum*
Genome Statistics	
Contigs	103
Genome Length	7412147
GC Content	60.722973
Contig L50	8
Contig N50	295871
Annotation Statistics	
tRNA	47
rRNA	3
CDS	7570
CDS Ratio	1.0212965
Hypothetical CDS	2102
Hypothetical CDS Ratio	0.37701452
PLFAM CDS	7004
PLFAM CDS Ratio	0.92523116
Genome Quality	
Coarse Consistency	99.3
Fine Consistency	95.4
CheckM Completeness	100
Genome Quality	Good

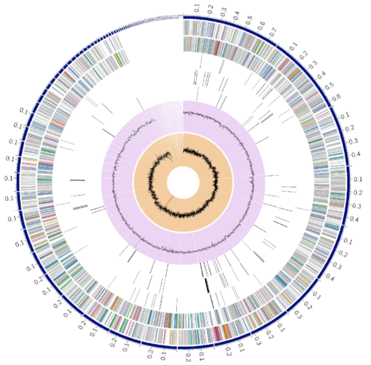

***Rhizobium leguminosarum* KB12**	
Length: 7412133bp, Contigs: 103	
General Info	
Genome ID	384.1013
Genome Name	*Rhizobium leguminosarum* KB12
Taxonomy Info	
Taxon ID	384
Kingdom	Bacteria
Phylum	Proteobacteria
Class	Alphaproteobacteria
Order	Hyphomicrobiales
Family	*Rhizobiaceae*
Genus	*Rhizobium*
Species	*Rhizobium leguminosarum*
Genome Statistics	
Contigs	103
Genome Length	7 412 133
GC Content	60.72313
Contig L50	8
Contig N50	295871
Annotation Statistics	
tRNA	47
rRNA	3
CDS	7567
CDS Ratio	1.0208937
Hypothetical CDS	2104
Hypothetical CDS Ratio	0.37729615
PLFAM CDS	7000
PLFAM CDS Ratio	0.9250694
Genome Quality	
Coarse Consistency	99.3
Fine Consistency	95.4
CheckM Completeness	100
Genome Quality	Good

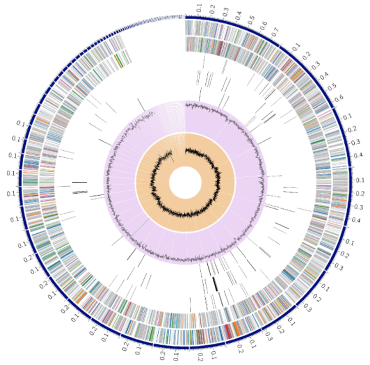

Supplementary Figure S3. A concise summary of the sequencing and contig assembly of the KB7, KB8, KB12, KC4, and KC5 genomes. The visual representation is divided into several sections and includes key information about each genome assembly. In the lower panel, individual rings represent (from outside to inside): genes on the leading strand, genes on the lagging strand, genes for non-coding RNAs, genes with homology to known antibiotic resistance genes, CDS with homology to known virulence factors, %GC plot, and GC skew plot.

A)

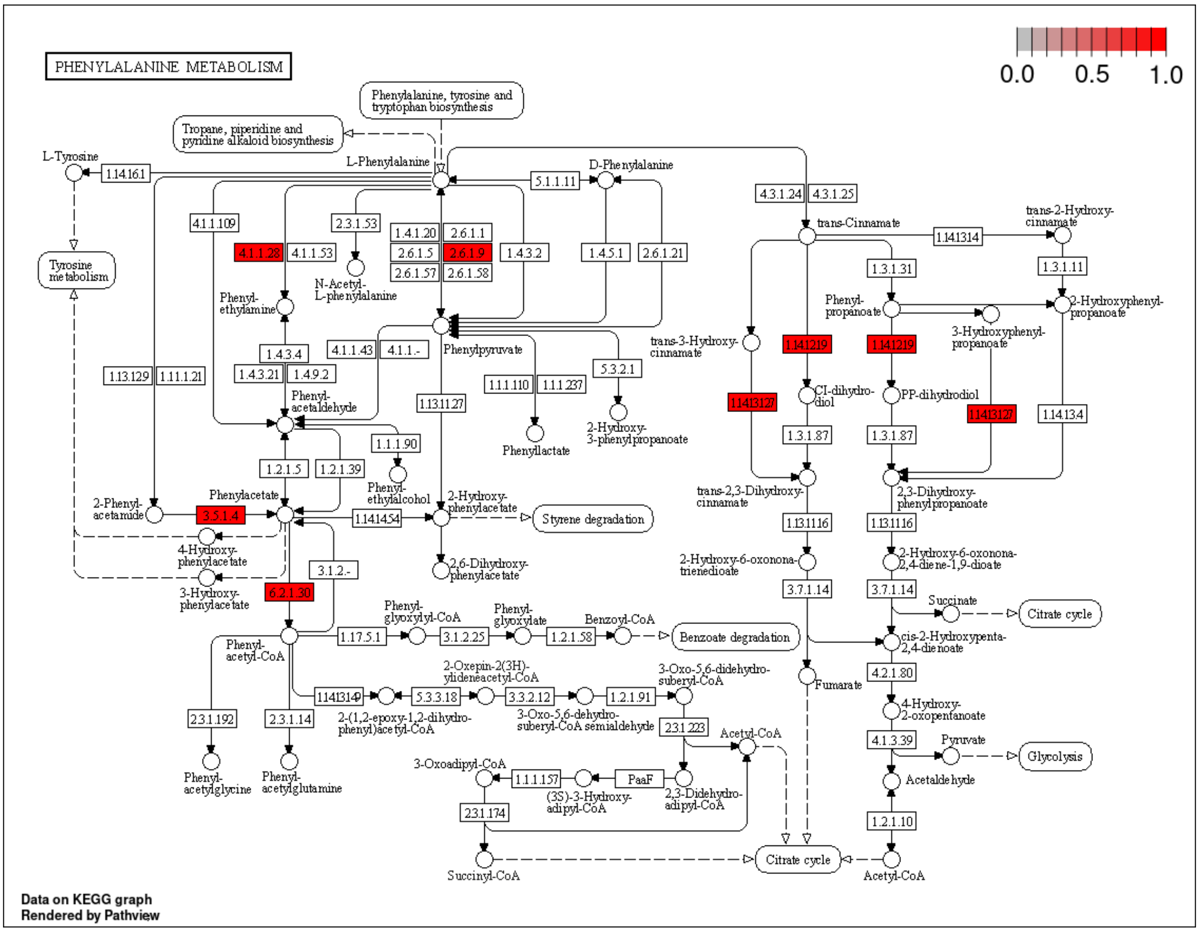

B)

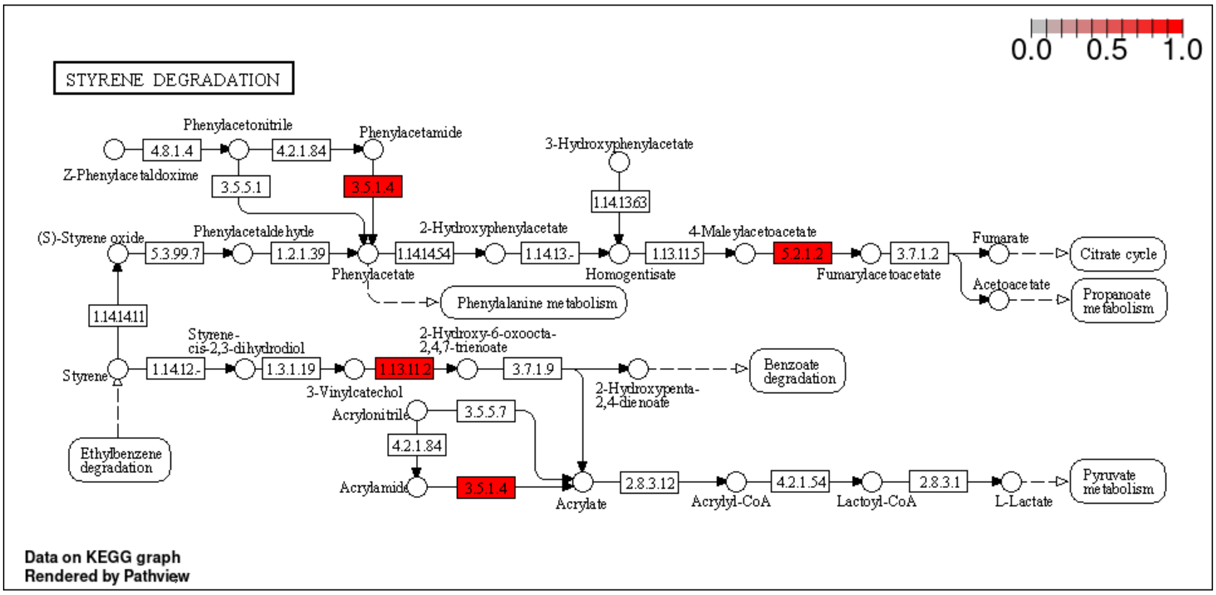
0.0 0.5 1.0
STYRENE DEGRADATION
Phenylacetonitrile
Phenylacetamide
3-Hydroxyphenylacetate
4.8.1.4
4.2.1.84
Z-Phenylacetaldoxime
3.5.5.1
3.5.1.4
1.14.13.63
(S)-Styrene oxide
Phenylacetaldehyde
2-Hydroxyphenylacetate
4-Maleylacetoacetate
Fumarate
5.3.99.7
1.2.1.39
1.14.14.54
1.14.13.-
1.13.11.5
5.2.1.2
3.7.1.2
Citrate cycle
Phenylacetate
Homogentisate
Fumarylacetoacetate
Acetoacetate
Propanoate metabolism
Phenylalanine metabolism
1.14.14.11
Styrene-cis-2,3-dihydrodiol
2-Hydroxy-6-oxoocta-2,4,7-trienoate
Styrene
1.14.12.-
1.3.1.19
1.13.11.2
3.7.1.9
Benzoate degradation
3-Vinylcatechol
2-Hydroxypenta-2,4-dienoate
Acrylonitrile
3.5.5.7
4.2.1.84
Ethylbenzene degradation
Acrylamide
3.5.1.4
2.8.3.12
4.2.1.54
2.8.3.1
Pyruvate metabolism
Acrylate
Acrylyl-CoA
Lactoyl-CoA
L-Lactate
Data on KEGG graph
Rendered by Pathview

C)

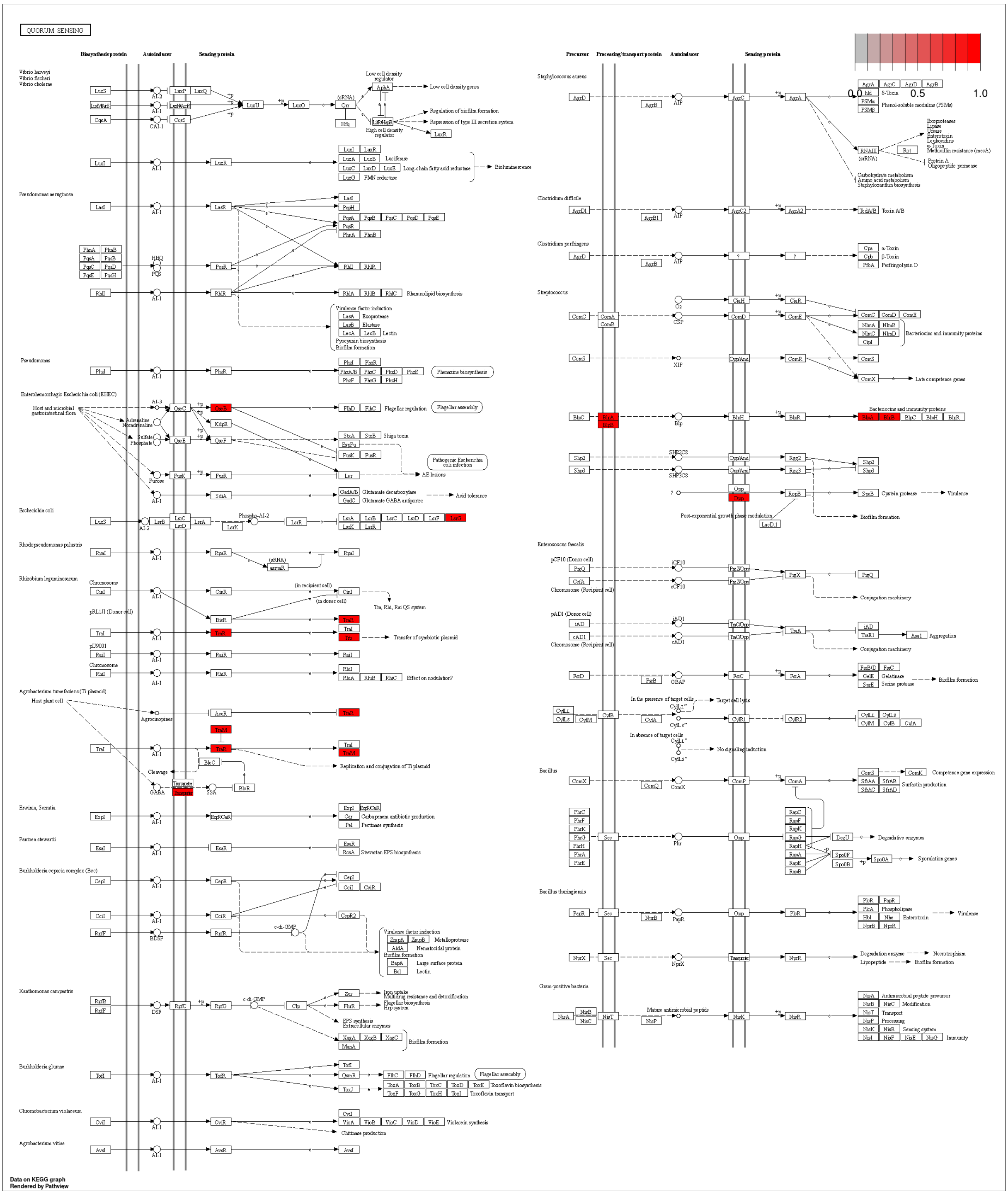

Supplementary Figure S4. Mapping the KB unique genes to the KEGG: A) phenylalanine metabolism (00360), B) styrene degradation (00643), C) quorum sensing (02024), pathway maps. Chemical compounds are represented as circles and gene products are represented as rectangles.

A)

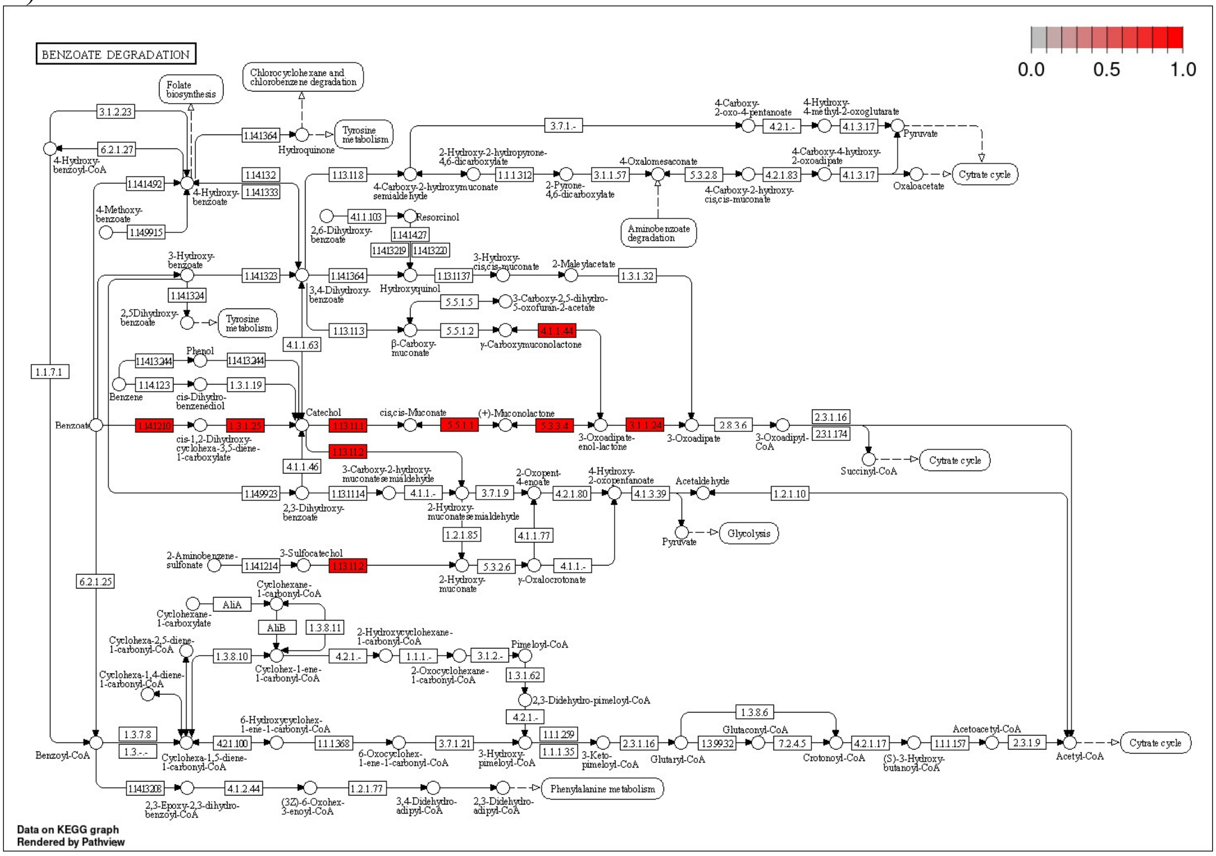
BENZOATE DEGRADATION
0.0
0.5
1.0
Folate biosynthesis
Chlorocyclohexane and chlorobenzene degradation
Tyrosine metabolism
Hydroquinone
4-Hydroxybenzoyl-CoA
4-Hydroxybenzoate
4-Methoxybenzoate
3.1.2.23
6.2.1.27
1.14.14.92
1.14.13.64
1.14.13.2
1.14.13.33
1.14.99.15
4-Carboxy-2-oxo-4-pentanoate
4-Hydroxy-4-methyl-2-oxoglutarate
3.7.1.-
4.2.1.-
4.1.3.17
Pyruvate
Oxaloacetate
Citrate cycle
2-Hydroxy-2-hydropyrone-4,6-dicarboxylate
4-Carboxy-2-hydroxymuconate semialdehyde
1.13.11.8
1.1.1.312
2-Pyrone-4,6-dicarboxylate
3.1.1.57
4-Oxalomesaconate
5.3.2.8
4-Carboxy-2-hydroxy-cis,cis-muconate
4.2.1.83
4-Carboxy-4-hydroxy-2-oxoadipate
Aminobenzoate degradation
2,6-Dihydroxybenzoate
4.1.1.103
Resorcinol
1.14.14.27
1.14.13.219
1.14.13.220
3-Hydroxybenzoate
1.14.13.23
1.14.13.24
1.14.13.64
3,4-Dihydroxybenzoate
Hydroxyquinol
1.13.11.37
3-Hydroxy-cis,cis-muconate
2-Maleylacetate
1.3.1.32
2,5-Dihydroxybenzoate
5.5.1.5
3-Carboxy-2,5-dihydro-5-oxofuran-2-acetate
1.13.11.3
5.5.1.2
4.1.1.44
β-Carboxymuconate
γ-Carboxymuconolactone
4.1.1.63
Phenol
1.14.13.244
Benzene
1.14.12.3
1.3.1.19
cis-Dihydrobenzenediol
Catechol
cis,cis-Muconate
(+)-Muconolactone
Benzoate
1.14.12.10
1.3.1.25
1.13.11.1
5.5.1.1
5.3.3.4
3-Oxoadipate-enol-lactone
3.1.1.24
3-Oxoadipate
2.8.3.6
3-Oxoadipyl-CoA
2.3.1.16
2.3.1.174
Succinyl-CoA
cis-1,2-Dihydroxycyclohexa-3,5-diene-1-carboxylate
1.13.11.2
4.1.1.46
3-Carboxy-2-hydroxymuconate semialdehyde
2-Oxopent-4-enoate
4-Hydroxy-2-oxopentanoate
Acetaldehyde
1.1.7.1
1.14.99.23
2,3-Dihydroxybenzoate
1.13.11.14
4.1.1.-
3.7.1.9
4.2.1.80
4.1.3.39
1.2.1.10
2-Hydroxymuconatesemialdehyde
1.2.1.85
4.1.1.77
Pyruvate
Glycolysis
2-Aminobenzenesulfonate
1.14.12.14
3-Sulfocatechol
2-Hydroxymuconate
5.3.2.6
γ-Oxalocrotonate
6.2.1.25
Cyclohexane-1-carboxylate
AliA
Cyclohexane-1-carbonyl-CoA
AliB
1.3.8.11
2-Hydroxycyclohexane-1-carbonyl-CoA
Cyclohexa-2,5-diene-1-carbonyl-CoA
1.3.8.10
Cyclohex-1-ene-1-carbonyl-CoA
4.2.1.-
1.1.1.-
2-Oxocyclohexane-1-carbonyl-CoA
3.1.2.-
Pimeloyl-CoA
1.3.1.62
Cyclohexa-1,4-diene-1-carbonyl-CoA
2,3-Didehydro-pimeloyl-CoA
6-Hydroxycyclohex-1-ene-1-carbonyl-CoA
4.2.1.-
1.3.8.6
Benzoyl-CoA
1.3.7.8
1.3.-.-
4.2.1.100
Cyclohexa-1,5-diene-1-carbonyl-CoA
1.1.1.368
6-Oxocyclohex-1-ene-1-carbonyl-CoA
3.7.1.21
3-Hydroxypimeloyl-CoA
1.1.1.259
1.1.1.35
3-Ketopimeloyl-CoA
2.3.1.16
Glutaryl-CoA
1.3.99.32
Glutaconyl-CoA
7.2.4.5
Crotonoyl-CoA
4.2.1.17
(S)-3-Hydroxybutanoyl-CoA
1.1.1.157
Acetoacetyl-CoA
2.3.1.9
Acetyl-CoA
Citrate cycle
1.14.13.208
2,3-Epoxy-2,3-dihydrobenzoyl-CoA
4.1.2.44
(3Z)-6-Oxohex-3-enoyl-CoA
1.2.1.77
3,4-Didehydroadipyl-CoA
2,3-Didehydroadipyl-CoA
Phenylalanine metabolism
Data on KEGG graph
Rendered by Pathview

B)

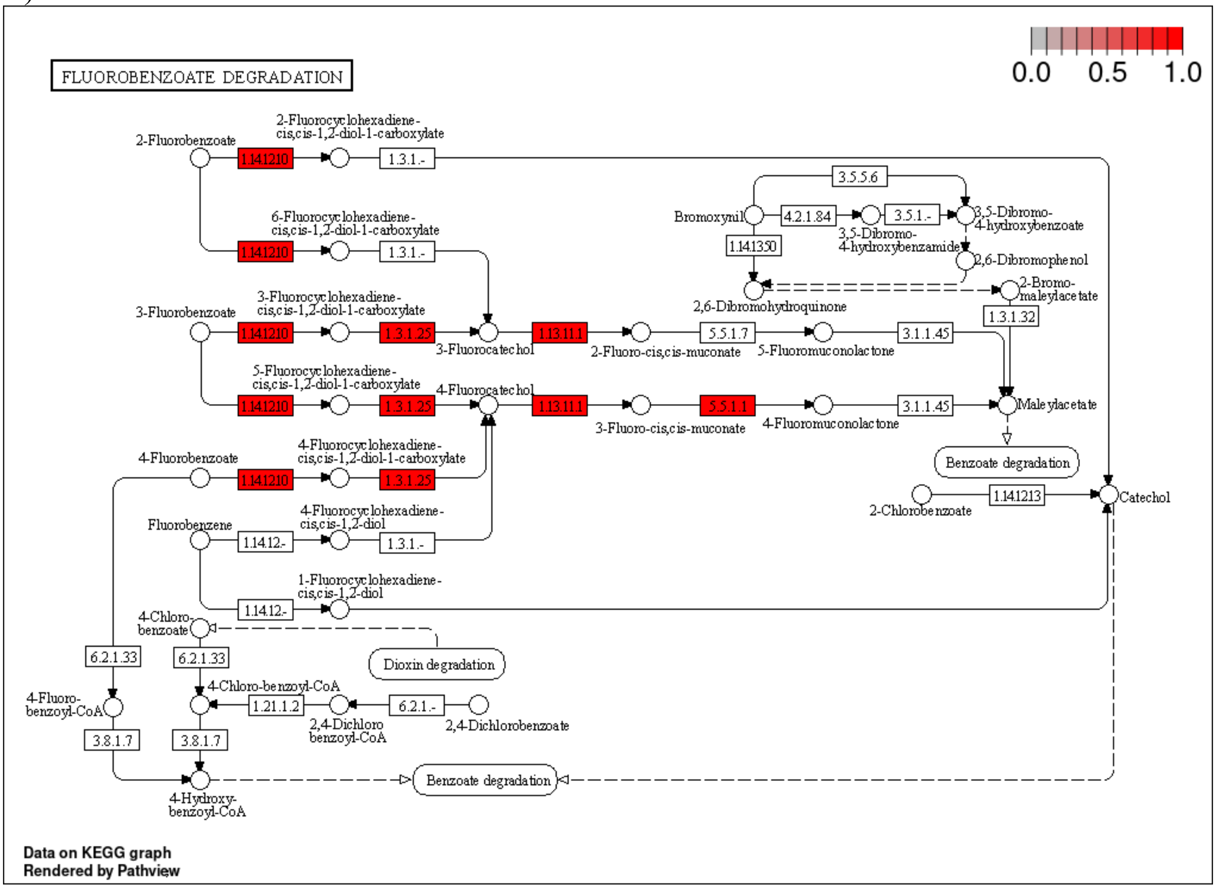
FLUOROBENZOATE DEGRADATION
0.0
0.5
1.0
2-Fluorobenzoate
2-Fluorocyclohexadiene-cis,cis-1,2-diol-1-carboxylate
1.14.12.10
1.3.1.-
6-Fluorocyclohexadiene-cis,cis-1,2-diol-1-carboxylate
1.14.12.10
1.3.1.-
3.5.5.6
Bromoxynil
4.2.1.84
3.5.1.-
3,5-Dibromo-4-hydroxybenzoate
1.14.13.50
3,5-Dibromo-4-hydroxybenzamide
2,6-Dibromophenol
2-Bromomaleylacetate
2,6-Dibromohydroquinone
1.3.1.32
3-Fluorobenzoate
3-Fluorocyclohexadiene-cis,cis-1,2-diol-1-carboxylate
1.14.12.10
1.3.1.25
3-Fluorocatechol
1.13.11.1
2-Fluoro-cis,cis-muconate
5.5.1.7
5-Fluoromuconolactone
3.1.1.45
5-Fluorocyclohexadiene-cis,cis-1,2-diol-1-carboxylate
1.14.12.10
1.3.1.25
4-Fluorocatechol
1.13.11.1
3-Fluoro-cis,cis-muconate
5.5.1.1
4-Fluoromuconolactone
3.1.1.45
Maleylacetate
Benzoate degradation
4-Fluorobenzoate
4-Fluorocyclohexadiene-cis,cis-1,2-diol-1-carboxylate
1.14.12.10
1.3.1.25
2-Chlorobenzoate
1.14.12.13
Catechol
Fluorobenzene
4-Fluorocyclohexadiene-cis,cis-1,2-diol
1.14.12.-
1.3.1.-
1-Fluorocyclohexadiene-cis,cis-1,2-diol
1.14.12.-
4-Chlorobenzoate
Dioxin degradation
6.2.1.33
6.2.1.33
4-Chloro-benzoyl-CoA
1.21.1.2
6.2.1.-
4-Fluorobenzoyl-CoA
2,4-Dichlorobenzoyl-CoA
2,4-Dichlorobenzoate
3.8.1.7
3.8.1.7
4-Hydroxybenzoyl-CoA
Benzoate degradation
Data on KEGG graph
Rendered by Pathview

C)

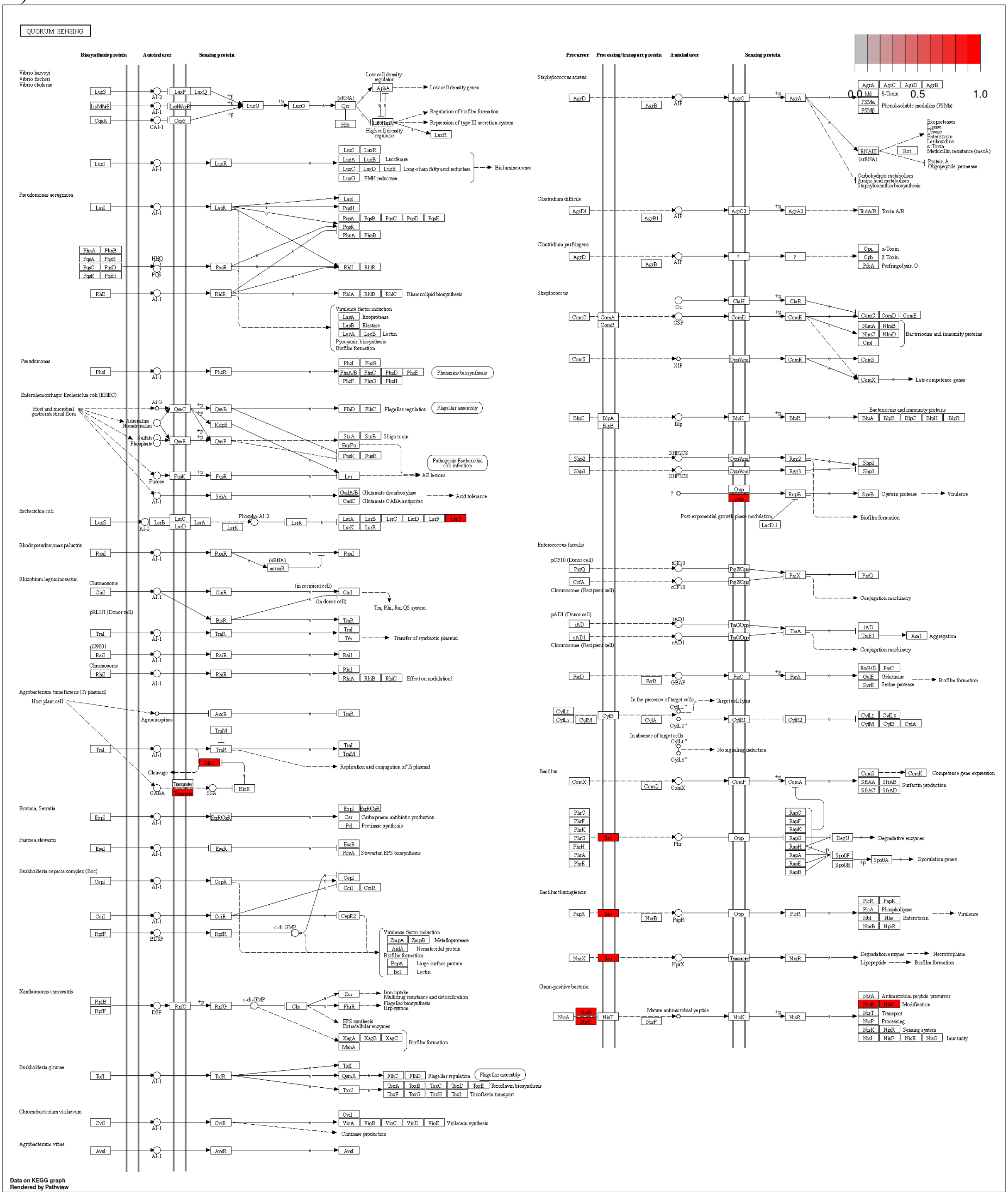

Supplementary Figure S5. Mapping the KC unique genes to the KEGG A) benzoate degradation (00362), B) fluorobenzonate degradation (00364), C) quorum sensing (02024) pathway maps. Chemical compounds are represented as circles and gene products are represented as rectangles.
